# A hybrid model based on general regression neural network and fruit fly optimization algorithm for forecasting and optimizing paclitaxel biosynthesis in *Corylus avellana* cell culture

**DOI:** 10.1186/s13007-021-00714-9

**Published:** 2021-02-05

**Authors:** Mina Salehi, Siamak Farhadi, Ahmad Moieni, Naser Safaie, Mohsen Hesami

**Affiliations:** 1 Department of Plant Genetics and Breeding, Faculty of Agriculture, Tarbiat Modares University, P.O. Box 14115-336, Tehran, Iran; 2 Department of Plant Pathology, Faculty of Agriculture, Tarbiat Modares University, Tehran, Iran; 3 Gosling Research Institute for Plant Preservation, Department of Plant Agriculture, University of Guelph, Guelph, ON Canada

**Keywords:** Anticancer, In vitro culture, Secondary metabolite, Optimization problem, Artificial intelligence, Mathematical modeling

## Abstract

**Background:**

Paclitaxel is a well-known chemotherapeutic agent widely applied as a therapy for various types of cancers. In vitro culture of *Corylus avellana* has been named as a promising and low-cost strategy for paclitaxel production. Fungal elicitors have been reported as an impressive strategy for improving paclitaxel biosynthesis in cell suspension culture (CSC) of *C. avellana*. The objectives of this research were to forecast and optimize growth and paclitaxel biosynthesis based on four input variables including cell extract (CE) and culture filtrate (CF) concentration levels, elicitor adding day and CSC harvesting time in *C. avellana* cell culture, as a case study, using general regression neural network-fruit fly optimization algorithm (GRNN-FOA) via data mining approach for the first time.

**Results:**

GRNN-FOA models (0.88–0.97) showed the superior prediction performances as compared to regression models (0.57–0.86). Comparative analysis of multilayer perceptron-genetic algorithm (MLP-GA) and GRNN-FOA showed very slight difference between two models for dry weight (DW), intracellular and extracellular paclitaxel in testing subset, the unseen data. However, MLP-GA was slightly more accurate as compared to GRNN-FOA for total paclitaxel and extracellular paclitaxel portion in testing subset. The slight difference was observed in maximum growth and paclitaxel biosynthesis optimized by FOA and GA. The optimization analysis using FOA on developed GRNN-FOA models showed that optimal CE [4.29% (v/v)] and CF [5.38% (v/v)] concentration levels, elicitor adding day (17) and harvesting time (88 h and 19 min) can lead to highest paclitaxel biosynthesis (372.89 µg l^−1^).

**Conclusions:**

Great accordance between the predicted and observed values of DW, intracellular, extracellular and total yield of paclitaxel, and also extracellular paclitaxel portion support excellent performance of developed GRNN-FOA models. Overall, GRNN-FOA as new mathematical tool may pave the way for forecasting and optimizing secondary metabolite production in plant in vitro culture.

## Background

Paclitaxel as a microtubule-stabilizing agent is widely used for the treatment of a vast range of cancers [1]. This natural source diterpene alkaloid, paclitaxel, is the most prosperous anticancer drug owing to its unique action mechanism [2]. Paclitaxel arrests the disassembly of the microtubule, and in this unique way inhibits mitosis and proliferation of cancerous cells [3, 4].

In vitro culture of hazel (*Corylus avellana*) has been named as a promising and low-cost strategy for paclitaxel production [5–13]. The advantages of paclitaxel production through *C. avellana* cell culture are that the establishment of its in vitro culture is more straightforward than that of *Taxus* [6–12], and also the response of hazel to genetic manipulation through *Agrobacterium* is likely more hopeful as compared to that of *Taxus* since *C. avellana* is a dicotyledonous plant [14]. Obtaining high-producing cell cultures is essential for producing secondary metabolites by way of plant in vitro culture [15]. Biosynthesizing bioactive compounds in plants are influenced by various factors [6–8, 16–19]. Fungal elicitors including cell extract (CE) and culture filtrate (CF) have been described as an impressive strategy for improving paclitaxel biosynthesis in cell suspension culture (CSC) of *C. avellana* [6, 7, 10–13]. Fungal elicitor type, concentration level and adding time as well as exposure time of cell culture to it (harvesting time) should be optimized to achieve the highest biosynthesis of paclitaxel in *C. avellana* CSC [6, 7, 10–13]. Precise analysis of the effects of these factors and their optimal selection would be a step forward to commercialize the bioprocess of *C. avellana* cells for paclitaxel mass production. Paclitaxel biosynthesis and its elicitation are the complex biological processes because they are influenced by multiple factors and their nonlinear interactions. Optimizing these mentioned factors by performing experiment is laborious, costly and time-consuming. Robust nonlinear computational methods can effectively predict the optimized conditions for multifactorial process [20, 21] such as paclitaxel biosynthesis.

Traditional modeling and forecasting methods including regression models display insignificant non-linear predictive and fitting ability [7, 12, 13]. Artificial intelligence (AI) is applied to address matters that cannot be clarified by traditional computational methods. Artificial neural networks (ANNs) are one of the main parts of AI discovering complex nonlinear relationships amongst input and output data [7, 13, 24–30]. Indeed, ANNs are brain-inspired systems that emulate human brain capability of sensing and thinking, in a simplified way, to processes information and identify patterns [31]. ANNs obtain their intelligence by discovering the relationships and patterns in data, and learn using experience [31].

General regression neural network (GRNN) developed by Specht [32] is a kind of radial basis function (RBF) networks, and one of the most popular neural networks. GRNN as a powerful regression method with a dynamic network structure can successfully solve problems with extremely difficult and unknown solution in various fields [33–39]. GRNN displays strong non-linear mapping capability, high fault tolerance, high robustness in the solution of complex problems, very fast network training speed, ease of implementation and simplicity of network structure [32, 40]. It is highly regretful that GRNN has not been used to model secondary metabolite biosynthesis in plant in vitro culture.

Smoothing (spread) parameter (σ) in GRNN architecture has an important effect on predicting performance [41]. Indeed, the generalization capability of GRNN model depends on smoothing parameter. Intelligent optimization algorithms including fruit fly optimization algorithm (FOA) [42] was applied to determine parameters for predicting models.

Fruit Fly optimization algorithm or fly optimization algorithm (FOA) presented by Pan [43] is a new evolutionary optimization algorithm inspired from food finding behavior of fruit fly. The advantages of FOA are easy computational process, relatively simple and short program code and ease of understanding. So, this research attempted to apply FOA to automatically determine smoothing factor value of GRNN for enhancing predicting accuracy, and also optimize factors “CE and CF concentration levels, adding day of fungal elicitor and CSC harvesting time” for maximum paclitaxel biosynthesis and secretion in *C. avellana* cell culture treated with fungal elicitors.

## Results

### General regression neural network-fruit fly optimization analysis

**Fig. 1:**
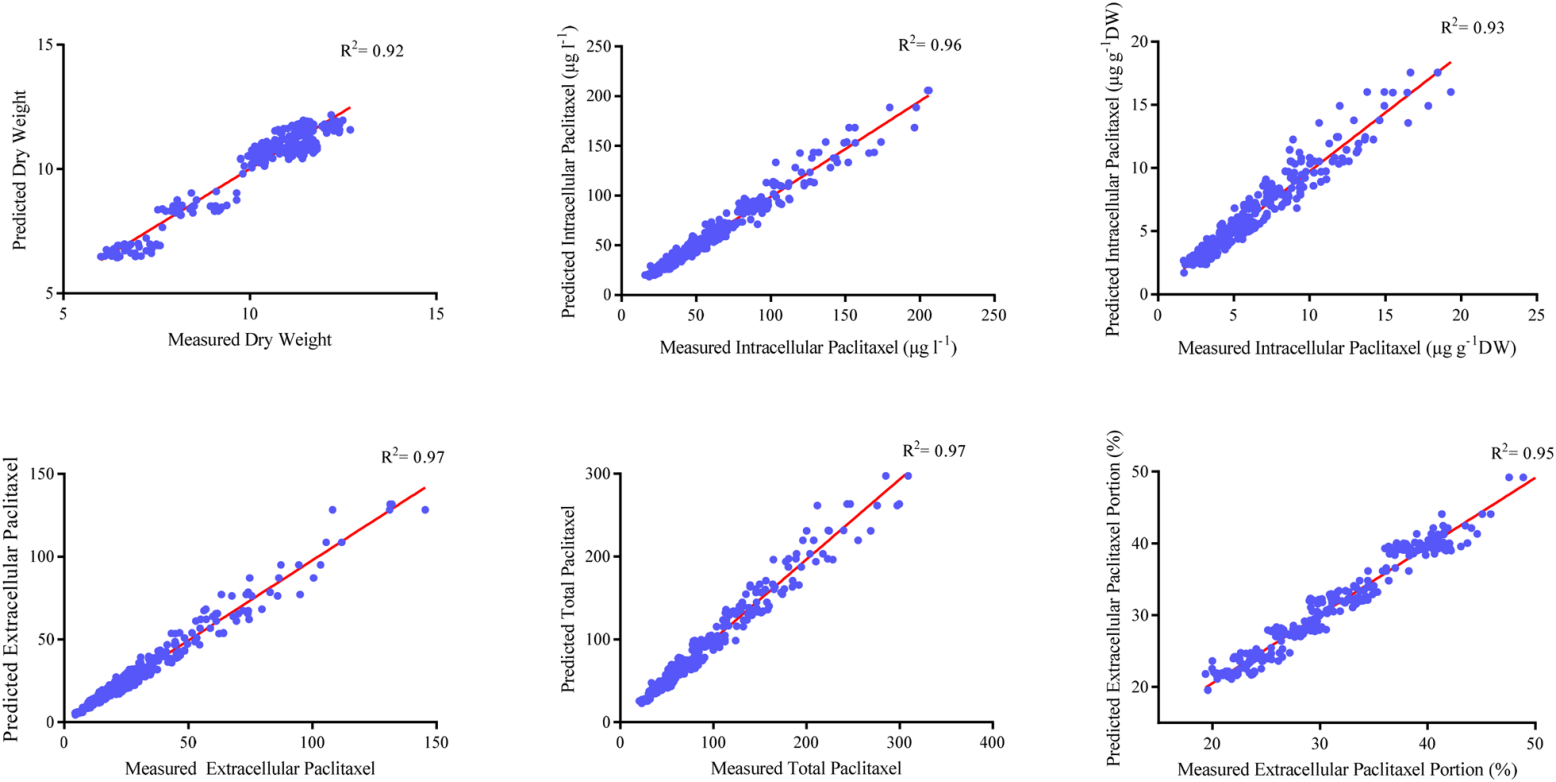
Scatter plot of actual data against predicted values of dry weight, intracellular (µg g^−1^DW), intracellular (µg l^−1^), extracellular and total yield of paclitaxel, and extracellular paclitaxel portion in *Corylus avellana* cell cultures using general regression neural network-fruit fly optimization algorithm (GRNN-FOA) models in training subset. The solid line shows fitted simple regression line on scatter points

**Fig. 2:**
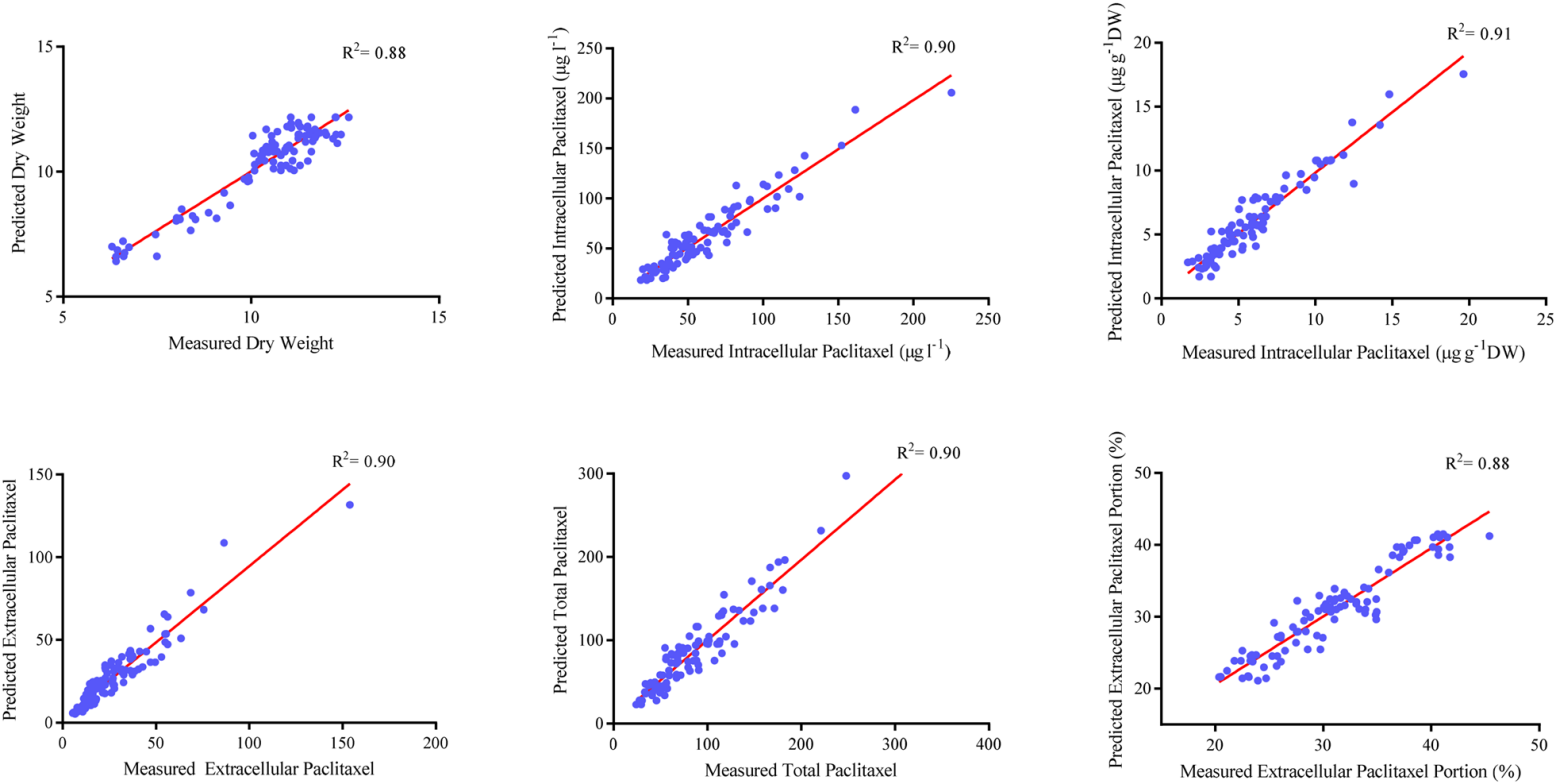
Scatter plot of actual data against predicted values of dry weight, intracellular (µg g^−1^DW), intracellular (µg l^−1^), extracellular and total yield of paclitaxel and extracellular paclitaxel portion in *Corylus avellana* cell cultures using general regression neural network-fruit fly optimization algorithm (GRNN-FOA) models in testing subset. The solid line shows fitted simple regression line on scatter points

Firstly, CE and CF concentration levels, elicitor adding day and CSC harvesting day were considered as input variables, and dry weight (DW), intracellular (µg g^−1^ DW), intracellular (µg l^−1^), extracellular and total yield of paclitaxel, and also extracellular paclitaxel portion as output variables. Afterwards, output variables were foretasted using developed GRNN-FOA models. The performance of developed GRNN-FOA models were evaluated by plotting the predicted values against the observed values of training (Fig. 1) and testing (Fig. 2) subsets. Great accordance between the predicted and observed values of DW, intracellular (µg g^−1^ DW), intracellular (µg l^−1^), extracellular and total yield of paclitaxel, and also extracellular paclitaxel portion was observed for both training and testing subset (Figs. 1, 2). Goodness-of-fit of developed GRNN-FOA models showed that they could accurately (R^2^ = 0.88, 0.90, 0.91, 0.90, 90 and 0.88) (Table 1) foretaste DW, intracellular (µg g^−1^ DW), intracellular (µg l^−1^), extracellular and total yield of paclitaxel as well as extracellular paclitaxel portion of testing subset, respectively, not used during training processes (Fig. 2).

**Table 1:** Statistics and information on general regression neural network-fruit fly optimization algorithm (GRNN-FOA) models for growth, paclitaxel biosynthesis and secretion in *Corylus avellana* cell culture

Measured factors	Training subsets			Test subsets		
	R^2^	RMSE	MBE	R^2^	RMSE	MBE
Dry weight (g l^−1^)	0.92	0.48	− 0.26 × 10^–15^	0.88	0.81	0.03
Intracellular paclitaxel (µg l^−1^)	0.96	7.38	− 2.22 × 10^–15^	0.90	10.99	0.65
Intracellular paclitaxel (µg g^−1^DW)	0.93	0.93	− 0.19 × 10^–15^	0.91	1.37	0.01
Extracellular paclitaxel (µg l^−1)^	0.97	4.43	− 0.47 × 10^–15^	0.90	6.93	− 0.09
Total yield of paclitaxel (µg l^−1^)	0.97	11.31	− 3.84 × 10^–15^	0.90	16.93	0.55
Extracellular paclitaxel portion (%)	0.95	1.46	− 0.76 × 10^–15^	0.88	2.88	− 0.23

*R*^*2*^ coefficient of determination, *RMSE* root mean square error, *MBE* mean bias error

### Sensitivity analysis of models

To rank input variables based on their relative importance in the model, variable sensitivity ratios (VSRs) were estimated using entire data lines (training and testing subsets). VSRs were obtained for each output variables (DW, intracellular (µg g^−1^ DW), intracellular (µg l^−1^), extracellular and total yield of paclitaxel, and also extracellular paclitaxel portion) regarding CE and CF concentration levels, elicitor adding day and CSC harvesting time (Table 2). Analysis of DW model indicated that DW of *C. avellana* cells was more sensitive to CSC harvesting time (VSR = 1.002), followed by elicitor adding day (VSR = 0.024), CE concentration level (VSR = 0.007) and CF concentration level (VSR = 0.005). Intracellular paclitaxel (µg g^−1^ DW) displayed more sensitivity to CE concentration level (VSR = 0.890), followed by CF concentration level (VSR = 0.675), elicitor adding day (VSR = 0.426) and CSC harvesting time (VSR = 0.244). Intracellular paclitaxel (µg l^−1^) exhibited more sensitivity to CE concentration level (VSR = 0.746), followed by CF concentration level (VSR = 0.441), elicitor adding day (VSR = 0.408) and CSC harvesting time (VSR = 0.396). Extracellular paclitaxel showed more sensitivity to CSC harvesting day (VSR = 0.948), followed by CF concentration level (VSR = 0.752), CE concentration level (VSR = 0.286) and elicitor adding day (VSR = 0.189). Accordingly, total yield of paclitaxel exhibited more sensitivity to CE concentration level (VSR = 0.689), followed by CF concentration level (VSR = 0.604), CSC harvesting time (VSR = 0.202) and elicitor adding day (VSR = 0.094). Also, extracellular paclitaxel portion displayed more sensitivity to CSC harvesting time (VSR = 0.422), followed by elicitor adding day (VSR = 0.141), CE concentration level (VSR = 0.100) and CF concentration level (VSR = 0.062) (Table 2).

**Table 2:** Importance (according to the sensitivity analysis) and optimal levels of the different factors including cell extract (CE), culture filtrate (CF) concentration levels [% (v/v)], fungal elicitor adding day and harvesting time (day) for achieving maximum growth, paclitaxel biosynthesis and its secretion in *Corylus avellana* cell culture by the optimization analysis using FOA and genetic algorithm (GA) on developed general regression neural network-fruit fly optimization (GRNN-FOA) models

Criteria	Variable	Importance value (according to VSR^a^)	Optimal level		Output optimal	
			FOA	GA	FOA	GA
Dry weight (g l^−1^)	CE concentration level	0.0068	4.26	5.34	12.57	12.18
	CF concentration level	0.0053	0.54	0.71		
	Adding day	0.0237	16.33	15.62		
	Harvest time	1.0022	20.58	20.86		
Intracellular paclitaxel (µg g^−1^DW)	CE concentration level	0.8904	4.12	3.45	19.26	18.53
	CF concentration level	0.6751	5.84	5.68		
	Adding day	0.4257	15.72	16.97		
	Harvest time	0.2443	20.34	20.41		
Intracellular paclitaxel (µg l^−1^)	CE concentration level	0.7458	4.43	5.07	224.78	213.78
	CF concentration level	0.4406	5.69	5.46		
	Adding day	0.4083	16.09	16.79		
	Harvest time	0.3961	20.47	21.09		
Extracellular paclitaxel (µg l^−1)^	CE concentration level	0.2862	4.58	4.73	152.15	141.11
	CF concentration level	0.7519	4.71	5.06		
	Adding day	0.1893	15.91	16.19		
	Harvest time	0.9477	22.06	22.86		
Total yield of paclitaxel (µg l^−1^)	CE concentration level	0.6891	4.29	4.97	372.89	369.04
	CF concentration level	0.6043	5.38	5.01		
	Adding day	0.0943	17.00	16.53		
	Harvest time	0.2018	20.68	20.16		
Extracellular paclitaxel portion (%)	CE concentration level	0.1003	4.62	5.06	50.36	49.63
	CF concentration level	0.0622	4.91	4.97		
	Adding day	0.1409	16.59	17.03		
	Harvest time	0.4224	22.66	21.98		

^a^Relative indication of the ratio between the variable sensitivity error and the error of the model when all variables are available

### Model optimization

The optimization analysis on developed GRNN-FOA models was performed using fruit fly optimization algorithm to determine optimal levels of input variables for achieving maximum growth, paclitaxel biosynthesis and its secretion in *C. avellana* CSCs (Table 2). The optimization results showed that adding 4.8% (v/v) of CE:CF (89:11) containing 4.26% (v/v) CE and 0.54% (v/v) CF on 16th day, and harvesting CSC 102 h after elicitation could result in the maximum DW (12.57 g l^−1^) (Table 2). The highest content of intracellular paclitaxel (19.26 µg g^−1^ DW) may produce by adding 9.96% (V/V) of CE:CF (41:59) containing 4.12% (v/v) CE and 5.84% (v/v) CF on 16^th^ day, and harvesting CSC 110 h and 53 min after elicitation (Table 2). *C. avellana* cell culture exposed with 10.12% (v/v) of CE:CF (44:56) containing 4.43% (v/v) CE and 5.69% (v/v) CF on 16th day, and harvesting it 105 h and 7 min after elicitation may obtain the highest intracellular paclitaxel (224.78 µg l^−1^). Also, the results showed that the highest extracellular paclitaxel (152.15 µg l^−1^) can be produced by adding 9.29% (v/v) of CE:CF (49:51) containing 4.58% (v/v) CE and 4.71% (v/v) CF on 16th day, and harvesting CSC 147 h and 36 min after elicitation (Table 2). Additionally, CSC exposed with 9.67% (v/v) of CE:CF (44:56) containing 4.29% (v/v) CE and 5.38% (v/v) CF on 17th day, and harvesting it 88 h and 19 min after elicitation may obtain the highest total yield of paclitaxel (372.89 µg l^−1^) (Table 2). The results of GRNN-FOA model optimization displayed that adding 9.53% (v/v) of CE:CF (48:52) containing 4.62% (v/v) CE and 4.91% (v/v) CF on 17^th^ day, and harvesting CSC 145 h and 41 min after elicitation may lead to the highest extracellular paclitaxel portion (50.36) (Table 2).

GRNN-FOA was also linked to genetic algorithm (GA) to determine the optimal level of input variables for achieving maximum growth, paclitaxel biosynthesis and its secretion in *C. avellana* CSCs (Table 2). The optimization results of paclitaxel biosynthesis in GRNN-FOA model using GA showed that adding 6.05% (v/v) of CE:CF (88:12) containing 5.34% (v/v) CE and 0.71% (v/v) CF on 16th day, and harvesting CSC 125 h and 46 min after elicitation could result in the maximum DW (12.18 g l^−1^) (Table 2). Also, optimization results indicated that intracellular paclitaxel (18.53 µg g^−1^ DW) may produce by adding 9.13% (V/V) of CE:CF (38:62) containing 3.45% (v/v) CE and 5.68% (v/v) CF on 17th day, and harvesting CSC 82 h and 34 min after elicitation. *C. avellana* cell culture exposed with 10.53% (v/v) of CE:CF (48:52) containing 5.07% (v/v) CE and 5.46% (v/v) CF on 17th day, and harvesting it 103 h and 12 min after elicitation may obtain the highest total intracellular paclitaxel (213.78 µg l^−1^). Additionally, the results showed that the highest extracellular paclitaxel (141.11 µg l^−1^) can be produced by adding 9.79% (v/v) of CE:CF (48:52) containing 4.73% (v/v) CE and 5.06% (v/v) CF on 16th day, and harvesting CSC 160 h and 6 min after elicitation (Table 2). Also, cell culture exposed with 9.98% (v/v) of CE:CF (50:50) containing 4.97% (v/v) CE and 5.01% (v/v) CF on 17th day, and harvesting it 87 h and 7 min after elicitation may obtain the highest total yield of paclitaxel (369.04 µg l^−1^) (Table 2). The results of optimizing GRNN-FOA model using GA showed that adding 10.03% (v/v) of CE:CF (50:50) containing 5.06% (v/v) CE and 4.97% (v/v) CF on 17th day, and harvesting CSC 118 h and 48 min after elicitation may lead to the highest extracellular paclitaxel portion (49.63) (Table 2).

### Validation experiment

*C. avellana* cell culture exposed to 4.29% (v/v) CE and 5.38% (v/v) CF on 17th day, and harvesting it 88 h after elicitation (optimized input variables in GRNN-FOA model using FOA) produced 348.65 ± 36.8 µg l^−1^ paclitaxel.

## Discussion

Paclitaxel biosynthesis in *C. avellana* CSC treated with fungal elicitors is affected by the type, concentration level and adding day of fungal elicitors and also CSC harvesting time [6, 7, 10–13]. Forecasting the optimized value of these mentioned factors is highly promising and essential for paclitaxel biosynthesis improvement. However, the optimization of these factors by experimental studies is laborious, time-consuming, and costly. Paclitaxel biosynthesis is considered as complex biological process since it is affected by multiple factors in nonlinear ways [7, 13]. Therefore, the conventional computational methods are inefficient for modeling paclitaxel biosynthesis [7, 12, 13]. Some machine learning algorithms such as multilayer perceptron [13], genetic algorithm [7, 13], adaptive neuro-fuzzy inference system [13] have been successfully used for forecasting and optimizing paclitaxel biosynthesis. This is the first study for forecasting the optimal conditions for maximum paclitaxel biosynthesis in *C. avellana* CSC exposed to fungal elicitors using GRNN-FOA model. To accurately forecast the optimized values of effective factors (CE and CF concentration levels, elicitor adding day and CSC harvesting time) on paclitaxel biosynthesis in *C. avellana* CSC, using a trustworthy modeling system is essential.

In this study, GRNN-FOA modeling was used to evaluate the relationships among four studied factors “CE and CF concentration levels, elicitor adding time and CSC harvesting time” and the parameters “DW, intracellular, extracellular and total yield of paclitaxel and extracellular paclitaxel portion”, and also the possibility of forecasting of paclitaxel biosynthesis by the determined factors. Such mathematical predictions using GRNN-FOA model have not been described in this area.

Our results suggested that GRNN-FOA models could accurately forecast DW, intracellular paclitaxel (µg g^−1^ DW), intracellular paclitaxel (µg l^−1^), extracellular paclitaxel, total yield of paclitaxel and extracellular paclitaxel portion (R^2^ = 0.88, 0.90, 0.91, 0.90, 0.90 and 88, respectively) in testing subset (Fig. 1), not used in training process. Small bias values (Table 2) showed the high potential of GRNN-FOA models in forecasting output variables.

It is noteworthy that our group was previously used multivariate statistical methods including “stepwise regression, ordinary least squares regression, principal component regression and partial least squares regression [12]. Goodness-of-fit showed no difference regarding the accuracy of different regression models for all output variables, 0.67, 0.57, 0.62, 0.60 and 0.86 for DW, intracellular paclitaxel, extracellular paclitaxel, total yield of paclitaxel and extracellular paclitaxel portion, respectively for training subset [12]. The fit of regression models was presented by R^2^ for testing subset, suggesting the best-mentioned regression models can explain 67, 62, 68, 65 and 86% of the variability in DW, intracellular paclitaxel, extracellular paclitaxel, total yield of paclitaxel and paclitaxel extracellular portion, respectively, when they faced unseen data [12]. As shown in Table 1, the statistical values for GRNN-FOA models displayed higher prediction accuracy than regression models in previous study [12]. This finding was in line with the previous studies [7, 13] showing AI technology had the superior performances as compared to conventional modeling methods for forecasting growth and paclitaxel biosynthesis in *C. avellana* cell culture.

Additionally, multilayer perceptron-genetic algorithm (MLP-GA) was used to forecast growth and paclitaxel biosynthesis in *C. avellana* CSC treated with fungal elicitors [13]. Comparative analysis of MLP-GA [13] and GRNN-FOA (Table 1) showed very slight difference between two models for DW, intracellular and extracellular paclitaxel in testing subset, the unseen data. However, MLP-GA was slightly more accurate as compared to GRNN-FOA for total paclitaxel and extracellular paclitaxel portion in testing subset. R^2^ for GRNN-FOA (Table 1) vs. MLP-GA [13] were; DW = 0.89 vs. 0.90, intracellular paclitaxel = 0.90 vs. 0.89, extracellular paclitaxel = 0.90 vs. 0.92, total yield of paclitaxel = 0.90 vs. 0.95, and extracellular paclitaxel portion = 0.88 vs. 0.91.

**Fig. 3:**
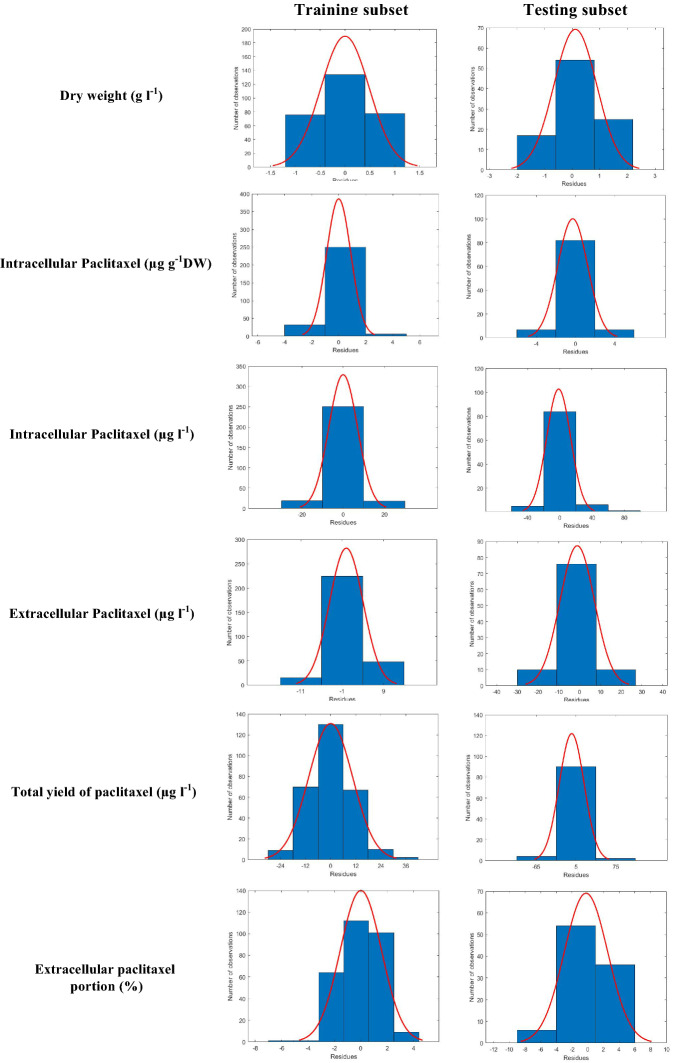
Histogram of residuals for general regression neural network-fruit fly optimization algorithm (GRNN-FOA) models developed for forecasting dry weight, intracellular (µg g^−1^DW), intracellular (µg l^−1^), extracellular and total yield of paclitaxel and extracellular paclitaxel portion in *Corylus avellana* cell cultures treated with fungal elicitors

As shown in Fig. 3, residual plots for all the developed GRNN-FOA models displayed a high density of points close to the origin and a low density of points away from the origin, and symmetric shape about the origin. Indeed, the residuals appear to behave randomly (normal distribution), it suggests that developed GRNN-FOA models for forecasting DW, intracellular paclitaxel (µg g^−1^ DW), intracellular paclitaxel (µg l^−1^), extracellular paclitaxel, total yield of paclitaxel and extracellular paclitaxel portion fit the data well.

The results of optimization analysis using “GA” and “FOA” on developed GRNN-FOA models displayed the slight difference in maximum growth and paclitaxel biosynthesis optimized by these optimization algorithms.

As previously mentioned, sensitivity analysis displayed that CE and CF concentration levels are the most important variables affecting total yield of paclitaxel (Table 2). Accordingly, CSC harvesting time and CF concentration level had the greatest effect on extracellular paclitaxel content (Table 2). The increment of paclitaxel secretion from the cells to culture medium decrease toxicity and feedback inhibition of paclitaxel [6, 13]. Paclitaxel secretion to culture medium undoubtedly makes easy extraction and the purification of it which is required for steady production of paclitaxel at the commercial level. Extracellular paclitaxel content is important for paclitaxel biosynthesis in continuous system. Sensitivity analysis displayed that CSC harvesting time is the most important factors affecting extracellular paclitaxel (Table 2). Paclitaxel biosynthesis is the complex biological processes which require the accurate techniques for modeling and optimization. GRNN-FOA has been efficiently used to solve problems with extremely difficult and unknown solution in various fields [40, 44–47].

Based on high forecasting accuracy of training and testing subsets (Figs. 1, 2) and also residual analysis (Fig. 3), it can be conclude that developed GRNN-FOA could precisely forecast DW, paclitaxel biosynthesis and secretion in *C. avellana* CSC. Additionally, the validation experiment revealed that GRNN-FOA hybrid method is an efficient method for forecasting and optimizing paclitaxel biosynthesis in *C. avellana* cell culture responding fungal elicitors.

In conclusion, this research applied GRNN-FOA for forecasting and optimizing paclitaxel biosynthesis in *C. avellana* cell culture treated with fungal elicitors for the first time. Great accordance between the predicted and observed values of DW, intracellular, extracellular and total yield of paclitaxel, and also extracellular paclitaxel portion support excellent performance of developed GRNN-FOA models. This research introduced GRNN-FOA as a new mathematical tool for forecasting and optimizing the complex systems including secondary metabolite biosynthesis in plant in vitro culture, paclitaxel biosynthesis in *C. avellana* CSC responding to fungal elicitors as a case study. Overall, GRNN-FOA could be useful as a strong method for forecasting and optimizing in various fields of plant systems.

## Methods

### Cell suspension culture

*C. avellana* CSC was established as described by Salehi et al. [8–11].

### Preparation of elicitors and elicitation experiment

Endophytic fungus applied in this research was a strain of *Camarosporomyces flavigenus*, HEF_17_, isolated from the leaf of *C. avellana* grown in Iran [13]. CE and CF were prepared as described previously [10]. For elicitation, 1.5 ± 0.1 g of *C. avellana* cells (fresh mass) was cultured in 100 ml flasks containing 30 ml of Murashige and Skoog (MS) medium supplemented with 2 mg l^−1^ 2,4-D and 0.2 mg l^−1^ BAP.

Three concentrations [2.5, 5 and 10% (v/v)] of fungal elicitors “CE:CF (100:0, 75:25, 50:50, 25:75, 0:100 v/v)”, and also mid (day 13) and late (day 17) log phase of *C. avellana* cell cultures were selected for adding fungal elicitors. Control received an equal volume of water (for CE)/potato dextrose broth (PDB) (for CF).

### Cell growth measurement

Cell dry weight (DW) was measured as cell growth [6–13].

### Quantification of paclitaxel

The extraction of intracellular and extracellular paclitaxel, and also HPLC analysis were performed with a procedure described by Salehi et al. [8–11].

## Experimental design

The experiment was planned based on completely randomized design (CRD) with factorial arrangement, three factors containing fungal elicitor type with 10 levels [(CE:CF (100:0, 75:25, 50:50, 25:75, 0:100 v/v) and water:PDB (100:0, 75:25, 50:50, 25:75, 0:100 v/v), elicitor concentration with three levels (2.5, 5, and 10% (v/v)], elicitor adding day with two levels (days 13 and 17), and three replicates. The cultures were harvested in two-day intervals after elicitation until 23rd day.

### Model development

Before testing machine learning algorithm, Box-Cox transformation [48] was used for normalizing the datasets. Also, principal component analysis (PCA) was applied to detect outliers; however, no outlier was detected in this case.

Five-fold cross-validation method with ten repetitions were used to calculate the prediction accuracy of all the tested models. Thus, we found the model with the best prediction on unknown data from the entire data set. The advantages of K-fold cross-validation are low computation time, low bias, every data dataset is used for both training (k − 1) and testing (1) subset.

### General regression neural network (GRNN) model

GRNN modeling was used to define the influences of CE and CF concentration levels, elicitor adding day and harvesting day on DW, paclitaxel biosynthesis (intracellular, extracellular and total) and extracellular paclitaxel portion.

**Fig. 4:**
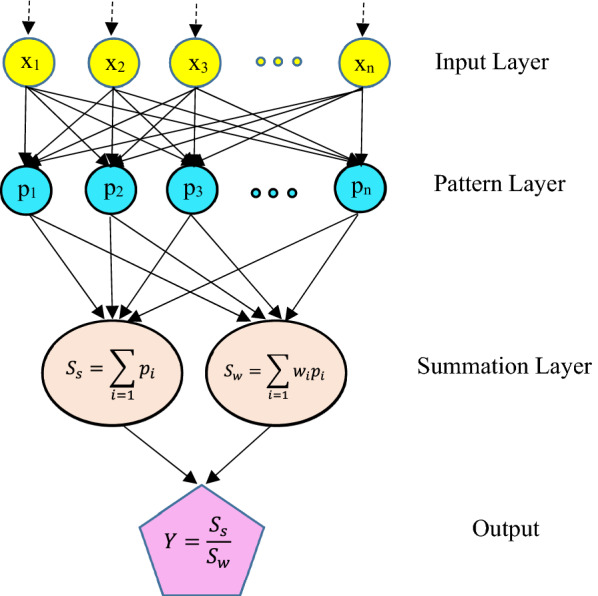
Schematic diagram of general regression neural network (GRNN) architecture

GRNN is established on a standard statistical method named Gaussian kernel regression [49]. As shown in Fig. 4, GRNN is made up of four layers including input, pattern, summation and output layers. Input layer (distribution unit) stores information as an input vector X, and is totally connected to pattern layer. The neurons of input layer, input neurons, feed input variables to all neurons on second layer (pattern unit). Pattern layer applies a non-linear transformation from input space to pattern one. Pattern neurons, the neurons in pattern layer, memorize the relation among input neuron and the proper response of pattern layer. Pattern Gaussian function “pi” given in Eq. (1) is applied to compute an output pi by a pattern neuron *i*.

1 $${p}_{i}=exp\left[-\frac{{\left(x-{x}_{i}\right)}^{T}(x-{x}_{i})}{2{\sigma }^{2}}\right] \left(i=\mathrm{1,2},\dots ,n\right),$$

where X denotes input variable, Xi is a specific training vector of pattern neuron *i*, and σ signifies smoothing parameter.

Summation neurons, the neurons in summation layer, pass on the outputs of pattern unit to third layer, summation unit. Third layer has two summations including simple summation (Ss) and weighted summation (Sw) while Ss (Eq. 2) computes the summation of all pattern layer outputs. Sw (Eq. 3) computes weighted sum of pattern layer outputs, where w_i_ is interconnection weight of pattern neuron *i* to summation layer.

2 $${S}_{s}=\sum_{i=1}{p}_{i}.$$

3 $${S}_{w}=\sum_{i=1}{w}_{i}{p}_{i}.$$

Then, summation layer feed both Ss (numerator) and Sw (denominator) to output layer. Output layer computes output Y of GRNN model by dividing summation layer outputs (Eq. 4).

4 $${Y=S}_{s}/{S}_{w}.$$

Smoothing parameter “σ” is only parameter that needs to be defined in GRNN model. This research applied fruit fly optimization algorithm (FOA) to automatically determine appropriate smoothing parameter value in GRRN model.

### Fruit fly optimization algorithm (FOA)

FOA was used (1) to determine appropriate value of smooth parameter (σ), and (2) to optimize the values of input variables (CE and CF concentration, elicitor elicitor adding day and CSC harvesting day) in developed GRNN-FOA models for maximum paclitaxel biosynthesis and its secretion.

**Fig. 5:**
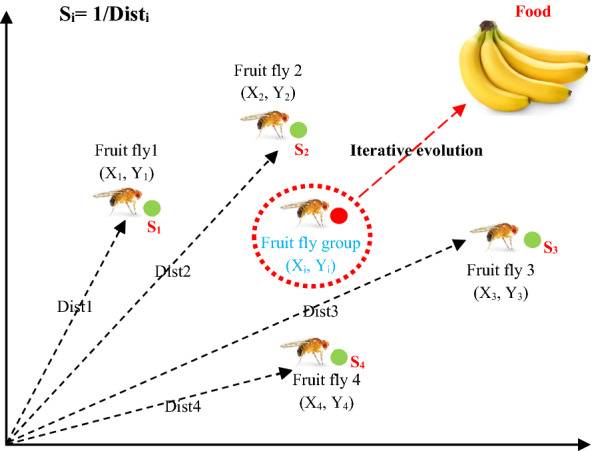
Food searching iterative process of fruit fly group

FOA is a new intelligence method inspired from food searching behavior of fruit fly which can find global optimal solution [43]. Food searching process of fruit fly includes two steps: (1) fruit fly detects the food location using osphresis organ and flies towards it, (2) when fruit fly gets close to the food source, the sensitive vision is likewise applied for detecting source and fruit flies flocking location, and fly towards that direction. Food finding iterative behavior of fruit fly group is presented in Fig. 5.

The procedure of FOA for detecting the optimal values is described as follows.

Step 1. Randomly initialize FOA parameters including population size (sizepop), maximum iteration number (maxgen), location coordinate (LC) (X, Y) of fruit fly group, and flight distance range (FDR).

Step 2. Give the random distance and direction (Eq. 5) to an individual fruit fly such that they can detect the food by osphresis organ.

5 $${X}_{i}=X+{Random}\; Value,$$

$${Y}_{i}=Y+{Random}\; Value.$$

Step 3. Compute the distance of food location to the origin (Dist) (Eq. 6), smell concentration judgment value (S_i_) (Eq. 7), and smell concentration (Smelli) of individual fruit fly location by putting smell concentration judgment value (Si) into the smell concentration judgment function (fitness function) (Eq. 8). At last, determine the fruit fly with highest smell concentration (highest Smell_i_ value) (Eq. 9) among the fruit fly group:

6 $${Dist}_{i}=\sqrt{\left({X}_{i}^{2}+{Y}_{i}^{2}\right).}$$

7 $${S}_{i}=1/{Dist}_{i}.$$

8 $${Smell}_{i}=Function \left({S}_{i}\right).$$

9 $$\left[bestSmell\,bestIndex\right]=max \left({Smell}_{i}\right).$$

Step 4. Keep the highest smell concentration value (Eq. 10), and find fly location coordinate with highest smell concentration value (Eq. 11), and at this point, fruit fly group flies towards that location using vision. Enter iterative optimization until (1) current iteration numbers is less than maxgen (2) highest smell concentration is superior as compared to previous iterative one.

10 $$Smellbest=bestSmell.$$

11 $$X=X\left(bestIndex\right).$$

$$Y=Y\left(bestIndex\right).$$

**Fig. 6:**
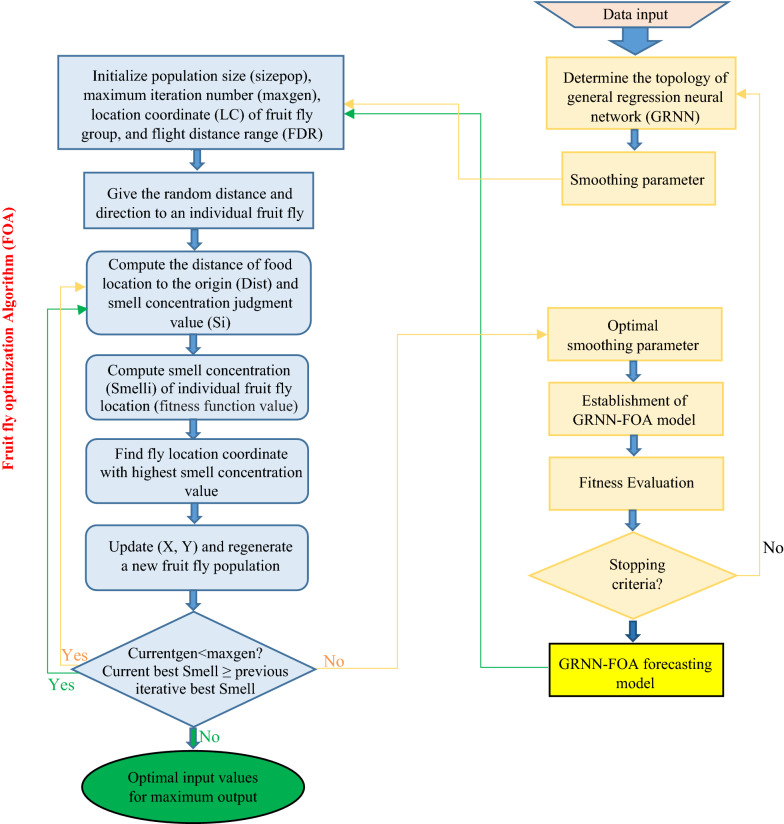
Flowchart of integrating general regression neural network (GRNN) with fruit fly optimization algorithm (FOA) for optimization of smoothing parameter in GRNN model, and also input values to achieve the highest amount of each output

The optimization procedure for searching appropriate value of smoothing parameter in GRNN model, and also optimal input variables for maximum paclitaxel biosynthesis through FOA in GRNN-FOA model is presented in Fig. 6. Maxgen of 100, sizepop of 10, LC of [0, 1] and FDR of [− 10, 10] [40] were set to establish the fittest GRNN structure, and also optimize input variables for maximum paclitaxel biosynthesis in GRNN-FOA model.

The performance of GRNN-FOA models is determined by three statistical criteria including root mean square error (RMSE) (Eq. 12), mean bias error (MBE) (Eq. 13) and coefficient of determination (R^2^) (Eq. 14).

12 $$RMSE= \sqrt{\left(\sum_{i=1}^{n}{\left({y}_{est}-{y}_{act}\right)}^{2}\right)/n,}$$

13 $$MBE= 1/n \sum_{i=1}^{n}\left({y}_{est}-{y}_{act}\right),$$

14 $${R}^{2}=1-\left(\sum_{i=1}^{n}{\left({y}_{est}-{y}_{act}\right)}^{2}/\sum_{i=1}^{n}{\left({y}_{act}-\stackrel{-}{y}\right)}^{2}\right),$$

where “y_act_” are the actual values, “y_est_” are the predicted values, and “n” is the number of data.

### Sensitivity analysis of the models

Sensitivity analysis was done on GRNN-FOA models to determine the importance degree of the factors (CE and CF concentration levels, elicitor adding day and harvesting time) on the model parameters (DW, paclitaxel biosynthesis and its secretion). The sensitivity of DW, paclitaxel biosynthesis (intracellular, extracellular and total yield) and extracellular paclitaxel portion was determined by the criteria including variable sensitivity error (VSE) value displaying the performance (RMSE) of GRNN-FOA model when that particular input variable is unavailable from the model. Variable sensitivity ratio (VSR) value was calculated as ratio of VSE and GRNN-FOA model error (RMSE value) when all input variables are available. The input variable with higher VSR was considered as higher important variable in model [7, 13, 50–52]. Finally, calculated VSR values were rescaled within range [0, 1] to make them more easily comparable.

The mathematical codes for the development and evaluation of GRNN-FOA and GRNN-FOA-GA models were written using MATLAB [53] software, and the graphs were made by GraphPad Prism 5 [54] software.

### Validation experiment

CE and CF concentration levels, elicitor adding day, and harvesting time of CSC optimized by FOA were tested to evaluate the efficiency of GRNN-FOA model for forecasting and optimizing paclitaxel biosynthesis in *C. avellana* cell culture responding to fungal elicitors.

## Data Availability

The datasets used and/or analyzed during the current study are available from the corresponding author on reasonable request.

## Abbreviations

CE: Cell extract
CF: Culture filtrate
CSC: Cell suspension culture
AI: Artificial intelligence
ANN: Artificial neural network
GRNN: General regression neural network
FOA: Fruit fly optimization algorithm
GA: Genetic algorithm
MLP: Multilayer perceptron
PDB: Potato dextrose broth
DW: Dry weight
VSR: Variable sensitivity ratio
